# Clinical and Molecular Correlates of Abnormal Changes in the Cerebellum and Globus Pallidus in Fragile X Premutation

**DOI:** 10.3389/fneur.2022.797649

**Published:** 2022-02-08

**Authors:** Jun Yi Wang, Jim Grigsby, Diego Placido, Hongjiang Wei, Flora Tassone, Kyoungmi Kim, David Hessl, Susan M. Rivera, Randi J. Hagerman

**Affiliations:** ^1^Center for Mind and Brain, University of California, Davis, Davis, CA, United States; ^2^Departments of Psychology and Medicine, University of Colorado Denver, Denver, CO, United States; ^3^Department of Psychology, University of California, Davis, Davis, CA, United States; ^4^School of Biomedical Engineering, Shanghai Jiao Tong University, Shanghai, China; ^5^Institute for Medical Robotics, Shanghai Jiao Tong University, Shanghai, China; ^6^Department of Biochemistry and Molecular Medicine, University of California Davis School of Medicine, Sacramento, CA, United States; ^7^The MIND Institute, University of California Davis Medical Center, Sacramento, CA, United States; ^8^Department of Public Health Sciences, University of California Davis School of Medicine, Sacramento, CA, United States; ^9^Department of Psychiatry and Behavioral Sciences, University of California Davis School of Medicine, Sacramento, CA, United States; ^10^Department of Pediatrics, University of California Davis School of Medicine, Sacramento, CA, United States

**Keywords:** eye-of-the-tiger sign, the MCP sign, fragile X premutation, brain iron accumulation, globus pallidus, cognitive impairment, neurodegenerative disorder, FXTAS

## Abstract

**Background:**

Fragile X premutation carriers (55–200 CGG triplets) may develop a progressive neurodegenerative disorder, fragile X-associated tremor/ataxia syndrome (FXTAS), after the age of 50. The neuroradiologic markers of FXTAS are hyperintense T2-signals in the middle cerebellar peduncle—the MCP sign. We recently noticed abnormal T2-signals in the globus pallidus in male premutation carriers and controls but the prevalence and clinical significance were unknown.

**Methods:**

We estimated the prevalence of the MCP sign and pallidal T2-abnormalities in 230 male premutation carriers and 144 controls (aged 8–86), and examined the associations with FXTAS symptoms, CGG repeat length, and iron content in the cerebellar dentate nucleus and globus pallidus.

**Results:**

Among participants aged ≥45 years (175 premutation carriers and 82 controls), MCP sign was observed only in premutation carriers (52 vs. 0%) whereas the prevalence of pallidal T2-abnormalities approached significance in premutation carriers compared with controls after age-adjustment (25.1 vs. 13.4%, *p* = 0.069). MCP sign was associated with impaired motor and executive functioning, and the additional presence of pallidal T2-abnormalities was associated with greater impaired executive functioning. Among premutation carriers, significant iron accumulation was observed in the dentate nucleus, and neither pallidal or MCP T2-abnormalities affected measures of the dentate nucleus. While the MCP sign was associated with CGG repeat length >75 and dentate nucleus volume correlated negatively with CGG repeat length, pallidal T2-abnormalities did not correlate with CGG repeat length. However, pallidal signal changes were associated with age-related accelerated iron depletion and variability and having both MCP and pallidal signs further increased iron variability in the globus pallidus.

**Conclusions:**

Only the MCP sign, not pallidal abnormalities, revealed independent associations with motor and cognitive impairment; however, the occurrence of *combined* MCP and pallidal T2-abnormalities may present a risk for greater cognitive impairment and increased iron variability in the globus pallidus.

## Introduction

The premutation of the fragile X mental retardation 1 (*FMR1*) gene, located on the long arm of the X chromosome, is an intermediate trinucleotide repeat expansion (55–200 CGG triplets) occurring in ~1:300 women and 1:850 men (1). *FMR1* premutation is associated with a spectrum of phenotypes that may affect premutation carriers at all ages (2). For example, common psychiatric problems affecting ~50% of premutation carriers include anxiety, attention-deficit/hyperactivity disorder, and social impairment during childhood (3–5), often with development of depression and obsessive compulsive symptoms or personality traits in adulthood (6, 7). During aging, premutation carriers show increased risk of developing fragile X-associated tremor/ataxia syndrome (FXTAS) with the penetrance increased from 17% in 50's to 75% for those aged ≥80 years (8). FXTAS is a progressive neurodegenerative disorder with variable presentation of core symptoms of intention tremor, cerebellar ataxia, parkinsonism, and impaired executive functioning (9–11). The primary neuroradiologic markers of FXTAS are hyperintense T2-signals in the middle cerebellar peduncle—the MCP sign (12). Other brain regions that may show T2-hyperintensities include the periventricular white matter, pons, corpus callosum, and deep white matter (13, 14). However, the MCP sign has been observed in five male premutation carriers without a clinical diagnosis of tremor or ataxia (15) and only 60% of men with FXTAS have the MCP sign on T2 scans (16). Further studies are needed to clarify the associations between the MCP sign and FXTAS clinical symptoms and identify additional brain regions that may contribute to FXTAS symptomology.

We recently saw abnormal hyperintense T2-signal changes in the globus pallidus in some carriers of the premutation, characterized by hyperintense T2-signals in the central region, surrounded by hypointense signals. Abnormal T2-signal changes in the globus pallidus have not previously been reported in individuals with the *FMR1* premutation, but we recently noted its presence in some carriers. Iron-related, T2-signal changes in the globus pallidus in premutation carriers may not be surprising given that neuropathological studies have revealed iron depositions in the choroid plexus and putamen (17, 18), and in the cerebellar cortex and dentate nucleus, in a subset of individuals with FXTAS (19). Hence, brain iron accumulation may represent a factor contributing to the neuropathology of FXTAS, at least in a significant portion of cases. We have observed significant atrophy and hypointense signal in diffusion weighted imaging (indicating increased iron depositions) in right globus pallidus among men with FXTAS (20). Globus pallidus is part of the basal ganglia-cerebellar-cortical network that is critical for motor, cognitive, and emotional processing (21).

The prevalence of the abnormal hyperintense T2-signals in the globus pallidus among premutation carriers and the clinical significance, alone or in combination with the MCP sign, remain uncertain. The current study therefore aims to: (1) estimate the prevalence of MCP and pallidal T2-abnormalities in men with normal and premutation *FMR1* alleles; (2) examine the relationships among MCP and pallidal T2-abnormalities, FXTAS symptoms, and iron content in the cerebellar dentate nucleus and globus pallidus; and (3) investigate the associations among MRI findings (i.e., T2-abnormalities and iron content) and *FMR1* molecular markers (i.e., CGG repeat length and mRNA level).

## Materials and Methods

### Research Participants

The study was conducted following procedures approved by the University of California Davis Institutional Review Board. Written informed consent was obtained from all participants. We collected all T2-weighted magnetic resonance imaging (MRI) scans from eligible male participants in previous and ongoing research studies at our site between 2007 and 2019. After excluding 15 scans with movement artifacts sufficient to interfere with the identification of pallidal T2-abnormalities, 614 scans were available from 374 males. These include 230 premutation carriers aged 8–86 years, of whom 110 had between 2 and 10 serial scans, and 144 healthy controls, aged 8–81 years, 22 of whom had 2–4 serial scans.

FXTAS diagnoses were made by trained physicians based on core FXTAS symptoms including intention tremor, cerebellar ataxia, cognitive impairment, and white matter hyperintensities (22). Tremor was rated using Clinical Rating Scale for Tremor (23), and ataxia was assessed using the International Cooperative Ataxia Rating Scale (24) and tandem gait testing. Executive functioning was assessed by the total score on the Behavioral Dyscontrol Scale (BDS), a nine-item instrument (range = 0–27) assessing the capacity for behavioral and attentional self-regulation (25). Psychological symptoms were evaluated using the Global Severity Index and Depression T-scores of Symptom Checklist-90-Revised (26).

### Molecular Genetic Data

Because both mosaicism and methylation of the *FMR1* CGG repeat element have been reported in a small percentage of male premutation carriers (27), CGG repeat size and methylation status were examined from genomic DNA isolated from peripheral blood lymphocytes, using PCR and Southern blot, as previously described (28–30). Percentage of methylation was measured by densitometry analysis (30). Male carriers with either one or two premutation alleles, and no evidence of methylation, were included in the study. For mosaic carriers (those with two *FMR1* alleles), CGG repeat size was averaged. Total RNA was purified from 2.5 mL peripheral blood and *FMR1* mRNA levels were measured by quantitative Real Time PCR using a 7,900 Sequence detector (PE Biosystems), as previously described (31).

### MRI Acquisitions

**Figure 1:**
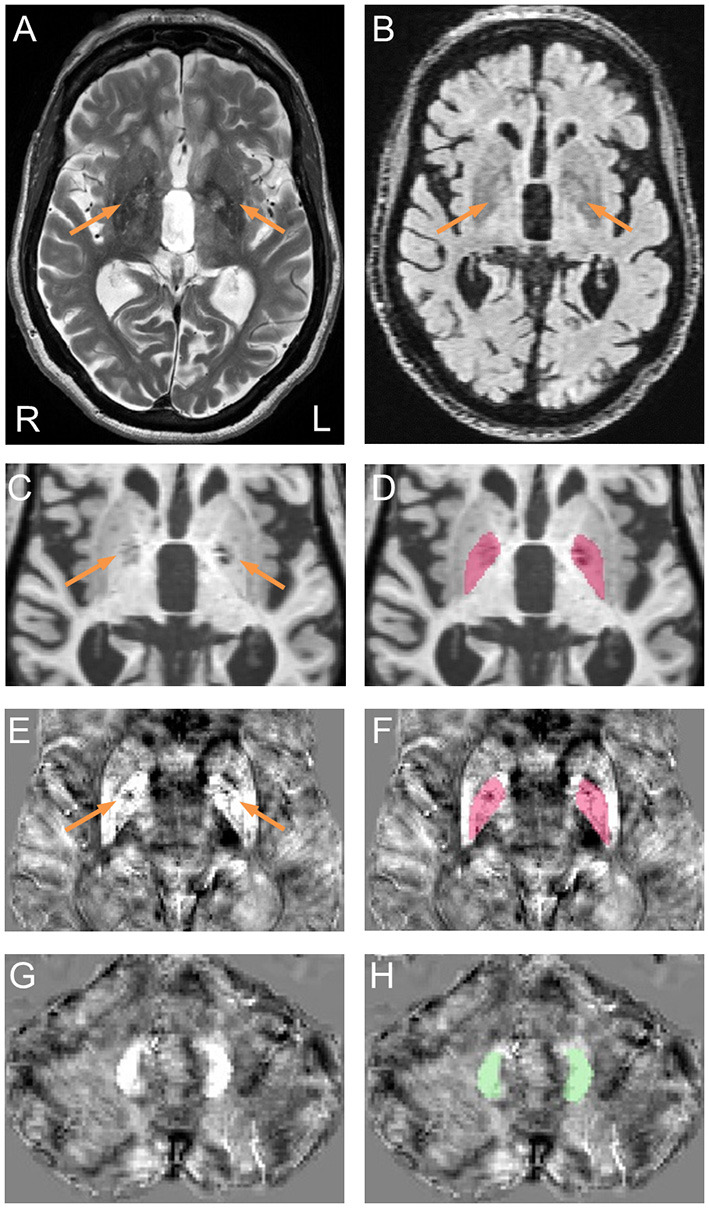
Representative pallidal T2-abnormalities and segmentation of the globus pallidus and dentate nucleus. **(A)** Pallidal T2-abnormalities (arrows) on a T2 scan acquired from a male premutation carrier who visited 5 times from age 62 to 74 years. The patient had action tremor and cerebellar ataxia that started at age 56 and “*Definite*” FXTAS diagnosis of at all visits. Behavioral Dyscontrol Scale scores decreased from 17 (visit 1) to 12 (visit 2) and to 10 (visits 3 and 4) and Symptom Checklist-90-Revised Global Severity Index scores increased from 56 (visit 1) to above 70 at visits 3, 4, and 5. **(B)** FLAIR scan shows hyperintensive signals in the central pallidal region (arrows) that correspond to the region showing hyperintensive signals in the T2 scan. **(C)** T1 scan shows bilateral signal loss in the globus pallidus (arrows). **(E,G)** QSM image co-registered to T1 scan, shows variation in susceptibility in the globus pallidus [**(E)**, arrows] but not in the dentate nucleus **(G)**. **(D,F)** Segmentation of the globus pallidus on T1 **(D)** and QSM **(F)**. **(H)** Segmentation of the dentate nucleus on QSM.

MRI scans were acquired on a Siemens Trio 3T MRI scanner (Siemens Medical Solutions, Erlangen, Germany), with either an 8-channel (167 scans) or 32-channel head coil (447 scans). Standard TSE T2-weighted scans or proton density-T2 scans with voxel size of 0.6 × 0.6 × 3.0 mm^3^ were utilized for detection of hyperintensive signals in both MCP and globus pallidus (Figure 1A). We carefully distinguished the abnormal hyperintensities from hyperintensities associated with enlarged perivascular spaces, which appeared as either scattered thin linearly-shaped hyperintensities throughout the globus pallidus or oval-shaped hyperintensities, typically <5 mm in diameter, with a smooth boundary inferior to the globus pallidus. All images included in quantitative analyses were acquired using the 32-channel head coil. T1-weighted scans were obtained using the standard magnetization-prepared rapid gradient echo sequence. For iron quantification, the 3D T2^*^-weighted multi-echo gradient recalled echo sequence was acquired in 64 axial slices of 2 mm thickness (no gap), with field of view = 224 mm^2^, matrix size = 256 × 256, repetition time = 50 ms, echo time_1_/spacing/echo time_8_ = 4/5.7/44 ms, and flip angle = 25°.

### MRI Analyses

MRI preprocessing and globus pallidus segmentation were performed according to recently established protocol (32). Briefly, a multi-atlas likelihood fusion algorithm implemented in BrainGPS (33–35) was utilized for globus pallidus segmentation. Errors were corrected automatically using a machine-learning method, SegAdapter (36, 37), followed by manual correction using ITK-Snap (38) (Figures 1C,D).

Iron concentration was estimated on T2^*^-weighted scans by quantitative susceptibility mapping (QSM). We used the open-source software package, STI Suite (39), to generate QSM images. Briefly, magnitude images were used to generate the brain masks. Raw phase images were unwrapped using Laplacian-based phase unwrapping (40–42), and phase images at multiple echoes were averaged to enhance signal-to-noise ratio. Local tissue phase was obtained by removing background phase via 3D spherical mean value filtering (41). Lastly, the tissue phase map was processed using the streaking artifact reduction for QSM (STAR-QSM) algorithm to obtain susceptibility maps (43).

For magnetic susceptibility measures, QSM and T1 images were co-registered by rigid body registration using SPM12 (44) (Figures 1E–H). The dentate nucleus was segmented manually on QSM images using ITK-Snap (Figures 1G,H). Intra-rater reliability, measured using intraclass correlation coefficient for absolute agreement (45), reached 0.84/0.96 (*p* = 0.017/0.009) for dentate volume/QSM mean. Coefficient of variation (CV) for globus pallidus QSM was calculated after eroding the segmentation by one voxel to avoid signal fluctuations along the boundary using the fslmaths command from FSL (46). Whole-brain QSM mean was used to normalize QSM data by subtraction. To account for individual differences in cranial size, brain scaling factor—the determinant of the affine transformation of brain and skull images to the standard space—was obtained using the SIENAX program from FSL (47, 48).

Two raters (DP and JYW), blind to participants' *FMR1* status and focused on the globus pallidus region during rating, each judged the presence of T2-hyperintensities in the central region of the globus pallidus independently for all 614 T2-scans. The two raters agreed on 94.4% of the ratings, and reached consensus after discussion for the remaining 5.6% of scans. To confirm that the observed pallidal T2-abnormalities were not enlarged perivascular spaces, we examined fluid-attenuated inversion recovery (FLAIR) scans for those with the sign. The FLAIR scans were acquired using the standard sagittal acquisition with an image resolution of 0.95 × 0.95 × 1.9 mm^3^. All FLAIR scans displayed hyperintense signals in same central pallidal region showing the hyperintense T2-signals (Figure 1B) except for a few scans that exhibited similar signal intensities as the peripheral region. None of the FLAIR scans showed hypointense signals consistent with enlarged perivascular spaces. Presence of the MCP sign was judged by one blinded, experienced imager, JYW. A second rating of the MCP sign in 30 randomly selected scans revealed agreement of 96.7%.

### Statistical Analyses

Statistical analyses were performed with the open-source R package (http://www.r-project.org/). Results were expressed as mean ± standard deviation (SD) or standard error (SE) of mean for quantitative variables and percentage (%) for categorical variables. For participants' characteristics at their last visits, *t*-tests were performed to compare the means of controls and premutation carriers for continuous variables not affected by age. The associations between the presence of tremor, ataxia, and MCP (dichotomous outcome, yes or no) at the last visits and *FMR1*-allele type (dichotomous predictor, premutation or normal, hereafter referred to as group) were estimated by multivariable logistic regression with age as a covariate. For continuous outcome variables affected by age, multiple linear regression was used to estimate the association between each of the outcomes and *FMR1*-allele type with age as a covariate. Since the presence of pallidal T2-abnormalities (dichotomous outcome) did not change across visits in most participants (97.6%), the association between the presence of pallidal T2-abnormalities and *FMR1*-allele type was estimated at the most recent visits using a logistic regression model. We also tested whether there was effect modification by age in the association by including an interaction term of group and age in the logistic model. Results from the logistic regression models are presented as odds ratios (ORs) with 95% confidence intervals (CIs). For repeated measures analyses, mixed-effects models (49) were used to determine the main effects of *FMR1*-allele type (group) and pallidal and MCP T2-abnormalities on behavioral/molecular/MRI outcome measures with age as a covariate. Behavioral/molecular/MRI outcome measures included intention tremor, cerebellar ataxia, executive function, psychological symptoms, *FMR1* measures (*i.e.*, CGG repeat length and *FMR1* mRNA level), and iron content in the globus pallidus and dentate nucleus. We also tested whether there was effect modification by age in the associations between the pallidal and MCP signs and behavioral/molecular/MRI outcome measures by including an interaction term of pallidal T2-abnormalities/MCP sign-by-age in the mixed-effects models. The mixed-effects models also included group-by-pallidal T2-abnormalities/MCP sign interaction to assess whether the effects of pallidal T2-abnormalties/MCP sign differed by groups, age-by-group-by-pallidal T2-abnormalities/MCP sign interaction to evaluate whether the effects of pallidal T2-abnormalties/MCP sign differed by group and age, pallidal T2-abnormalities-by-MCP interaction to evaluate whether the effects of pallidal T2-abnormalties differed by the presence/absence of the MCP sign, and age-by-pallidal T2-abnormalities-by-MCP interaction to evaluate whether the effects of pallidal T2-abnormalties differed by the presence/absence of the MCP sign and age. The mixed-effects models included participant as a random variable to account for individual random effects. Results from the regression models are presented as coefficient estimate of parameter (β) with SE.

Anatomic volume (of the dentate nucleus and globus pallidus) was included as a covariate when comparing QSM means, to account for potential effects of the same amount of iron depositions in a smaller structure (due to atrophy). Model selection was carried out using a step-wise approach via likelihood ratio tests. The analysis of residuals was performed to validate the regression models and underlying assumptions using residual plots prior to statistical inference. The R package “rmcorr” was utilized to estimate the correlation between repeated measures of globus pallidus and dentate nucleus. For each statistical analysis, Benjamini-Hochberg false discovery rate (FDR) (50) was used to control a family-wise type 1 error rate at 5% for multiple testing of multiple outcome measures.

## Results

### Descriptive Statistics

All single and serial scans were utilized in the detection of pallidal T2-abnormalities. The youngest man showing the MCP sign was a 50-year-old premutation carrier and the youngest man showing the pallidal T2-abnormalities was a 47-year-old premutation carrier. Hence, subsequent analyses included only those aged ≥45 years (*N* = 257). Serial scans were available from 48.2% of the participants among whom 21/82 controls had 2–3 visits over an average interval of 4.79 years (SD = 1.33 years, range = 1.91–7.49 years), and 103/175 premutation carriers who had 2–10 visits over an average interval of 3.87 years (SD = 2.91 years, range = 0.8–11.4 years).

Table 1 shows summary statistics of participant characteristics at their last visits. Since the premutation carriers were older than the controls (66.6 ± 8.1 vs. 63.6 ± 8.4 years; *p* = 0.008), all statistical analyses for FXTAS motor, cognitive, and psychological symptoms were accounted for age. As expected for individuals having the risk of a movement disorder, the premutation carriers showed increased prevalence of intention tremor (odds ratio = 8.30; 95% CI = (4.2, 16.4); *p* < 0.001) and cerebellar ataxia compared to controls (odds ratio = 13.5; 95% CI = (5.71, 31.9); *p* <0.001). Executive functioning was assessed using BDS, on premutation carriers scored lower than controls (β = −3.14, SE = 0.72, *p* < 0.001). For psychological symptoms evaluated using the Symptom Checklist-90-Revised, the premutation group revealed both increased Global Severity Index (β = 4.86, SE = 1.71, *p* = 0.005) and depression scores (β = 4.41, SE = 1.77, *p* = 0.013) relative to the control group.

**Table 1:** Characteristics of research participants at their most recent visits (age ≥ 45 years).

**Groups**	**Controls**	**Premutation carriers**	***P* Values**
Age: mean (SD) [N]	63.6 (8.4) [82]	66.6 (8.1) [175]	0.008
Intention tremor: % [N]	21.4 [70]	71.2 [170]	<0.001
Cerebellar ataxia: % [N]	10.0 [70]	61.8 [170]	<0.001
BDS: mean (SD) [N]	22.1 (3.1) [68]	18.3 (5.8) [166]	<0.001
Psych. symptoms: mean (SD) [N]	52.3 (11.2) [61]	56.2 (11.3) [156]	0.005
Depression: mean (SD) [N]	53.6 (11.1) [61]	57.2 (11.9) [156]	0.013
MCP sign: % [N]	0 [82]	52.0 [175]	<0.001
Pallidus T2-abnormalities: % [N]	13.4 [82]	25.1 [175]	0.069
With both signs of pallidus and MCP: % [N]	0 [82]	16.0 [175]	<0.001
CGG repeat: mean (SD) [N]	28.9 (4.6) [80]	90.1 (20.3) [174]	<0.001
*FMR1* mRNA: mean (SD) [N]	1.34 (0.39) [79]	2.64 (0.80) [174]	<0.001

*SD, standard deviation; BDS, Behavioral Dyscontrol Scale; MCP, middle cerebellar peduncle; FMR1, fragile X mental retardation 1*.

### Only Premutation Carriers Show the MCP Sign but Prevalence of Pallidal T2-Abnormalities Is Not Significantly Higher in Premutation Carriers After Age-Adjustment

Pallidal T2-abnormalities were observed in 13.4% of controls and 25.1% of premutation carriers at their most recent visits. However, the prevalence was not significantly different between controls and premutation carriers after age-adjustment (odds ratio = 1.97; 95% CI = (0.95, 4.09); *p* = 0.069). In contrast, no controls displayed the MCP sign at any visits, while 52% of the premutation carriers showed the MCP sign and 16.0% showed both pallidal T2-abnormalities and MCP sign at their last visits (Table 1). The high prevalence of the pallidal T2-abnormalities in controls was unexpected. To further characterize the controls with the sign, clinical and molecular features of controls with and without the sign were compared at their last clinical visits. Only depression score was significantly lower in controls with the pallidal sign relative to controls without the sign at FDR < 0.05 (β = −10.9, SE = 3.79, *p* = 0.006) (Supplementary Table 1).

### Greater Impairment of Executive Function in Premutation Carriers With Both MCP and Pallidal T2-Abnormalities

**Figure 2:**
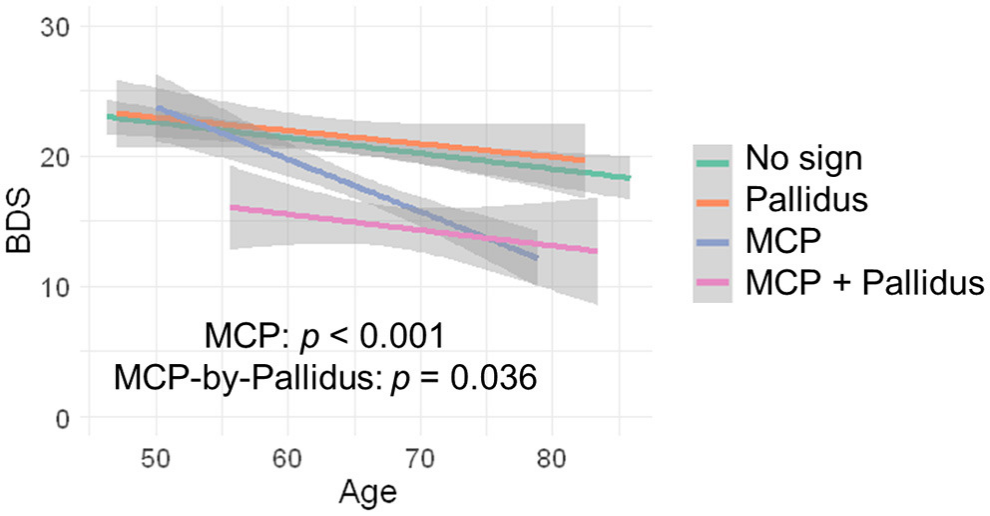
The effect of MCP sign and pallidal T2-abnormalities on executive function. Having the MCP sign is associated with low BDS scores and having both the pallidal T2-abnormalities and MCP sign is associated with even lower BDS scores.

Since no controls showed the MCP sign, only premutation carriers were included in analyses of the interaction between pallidal and MCP T2-abnormalities on FXTAS clinical symptoms. The MCP sign was significantly associated with both intention tremor and cerebellar ataxia in 170 premutation carriers (tremor/ataxia odds ratio = 16.0/3.64E + 07, *p* = 0.007/ <0.001) (Table 2). The effect of the signs on executive function was estimated including the data from all visits for 166 premutation carriers. The MCP sign demonstrated a significant negative effect on executive functioning after adjusting for age (MCP effect: β = −2.90, *p* < 0.001) (Table 2), and the presence of additional pallidal T2-abnormalities was associated with more impaired executive function (pallidal-by-MCP: β = −3.04, *p* = 0.036) (Figure 2). In contrast to the MCP sign, pallidal T2-abnormalities were not associated with core FXTAS motor symptoms such as tremor and ataxia in the controls or premutation carriers. Neither the pallidal nor MCP T2-abnormalities were correlated with psychological symptoms.

**Table 2:** The effect of T2-abnormalities in the MCP and globus pallidus on FXTAS clinical symptoms in premutation carriers.

**Comparisons**	***OR/β***	**95% CI**	***P***	**FDR**
**Intention tremor (N** **=** **170)**				
MCP (odds ratio)	16.0	(2.12, 120.6)	0.007^*^	0.012
**Cerebellar ataxia (N** **=** **170)**				
MCP (odds ratio)	3.64E + 07	(7.7E + 05, 1.7E + 10)	<0.001^*^	<0.001
**BDS (N** **=** **166)**				
Pallidus	1.41	(−0.72, 3.54)	0.20	0.20
MCP	−2.90	(−4.30, −1.49)	<0.001^*^	<0.001
MCP-by-pallidus	−3.04	(−5.86, −0.22)	0.036^*^	0.045

* *Significant at FDR 0.05*.

*OR, odds ratio; CI, confidence interval; FDR, false discovery rate; MCP, middle cerebellar peduncle*.

### Only the MCP Sign Shows a Correlation With CGG Repeat Length

Associations of the two signs with *FMR1* molecular measures (CGG repeat length and mRNA level) were analyzed at last visits for 174 premutation carriers. While no association was detected for the pallidal T2-abnormalities (CGG/mRNA: β = 4.85/0.11, SE = 3.05/0.11, *p* = 0.11/0.31), the MCP sign was positively associated with CGG repeat length (β = 14.3, SE = 2.45, *p* < 0.001). The MCP sign was observed in 91/140 (65%) premutation carriers with CGG repeat size >75, but was not present at any visits among 34 carriers with CGG repeat length ≤ 75. No significant associations between the MCP sign and *FMR1* mRNA level were detected (β = 0.18, SE = 0.10, *p* = 0.08).

### Presence of Combined MCP and Pallidal T2-Abnormalities Associated With Increased Iron Variability in the Globus Pallidus

**Figure 3:**
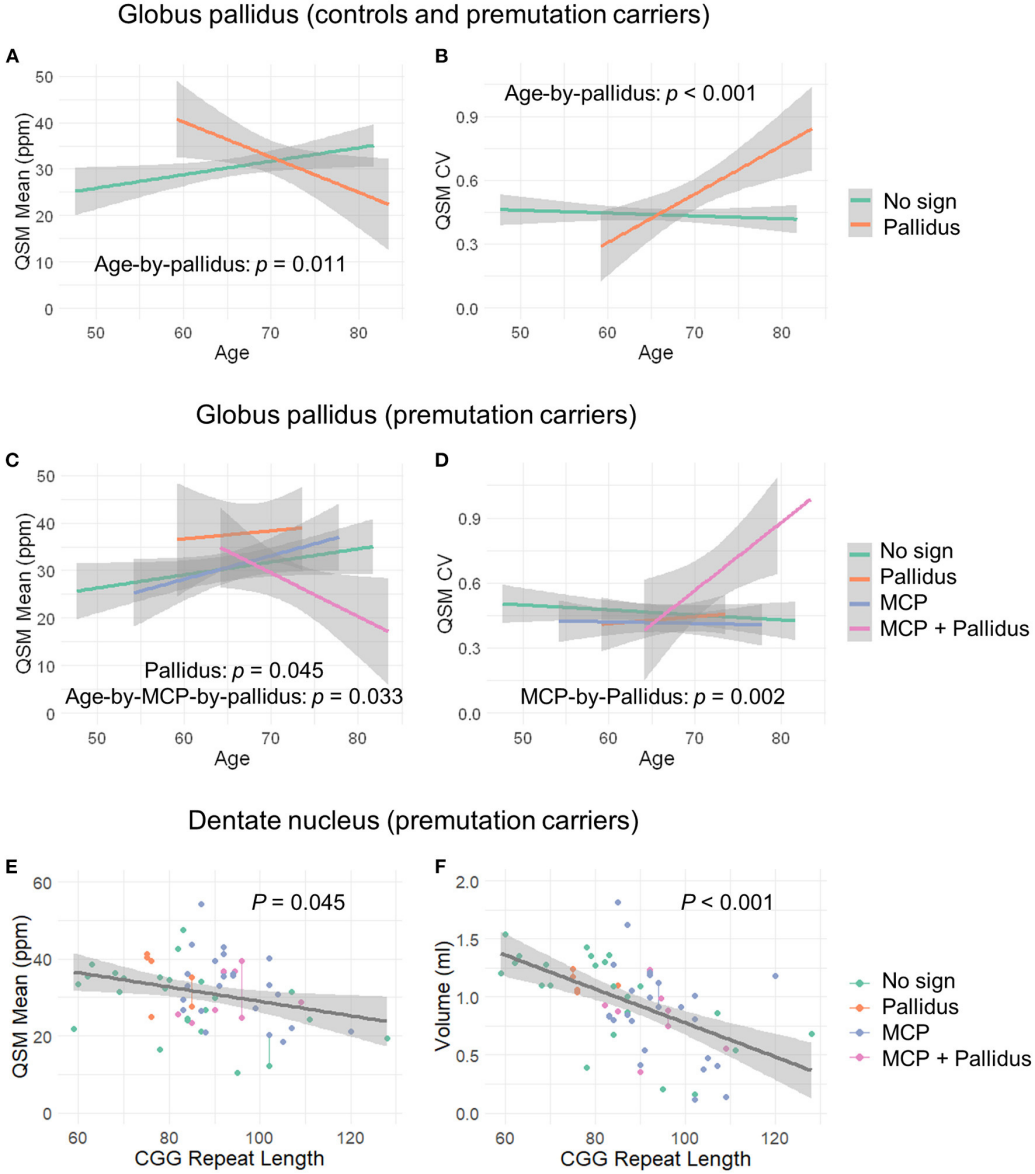
The effect of MCP sign, pallidal T2-abnormalities, and CGG repeat length on the globus pallidus and dentate nucleus. **(A)** Age-related decrease in iron content in the globus pallidus for controls and premutation carriers with pallidal T2-abnormalities in contrast to age-related increase in iron content for those without the sign. **(B)** Age-related increase in iron variability in the globus pallidus for controls and premutation carriers with pallidal T2-abnormalities in contrast to non-significant age-related changes for those without the abnormalities. **(C)** Premutation carriers with pallidal T2-abnormalities showed increased iron in the globus pallidus compared to premutation carriers without the abnormalities, and premutation carriers with both pallidal and MCP T2-abnormalities showed decreased iron with age in the globus pallidus in contrast to the remaining premutation carriers who exhibited increased iron with age. **(D)** Premutation carriers with both pallidal and MCP T2-abnormalities revealed increased iron variability in the globus pallidus relative to the remaining premutation carriers. However, the apparent age-by-group interaction was not significant. **(E)** Negative correlation between CGG repeat length and iron content in the dentate nucleus in the premutation carriers. **(F)** Negative correlation between CGG repeat length and volume of dentate nucleus in the premutation carriers.

QSM for quantifying iron content was available for a subset of the participants, including 9 controls and 61 premutation carriers (76 scans, only 6 premutation carriers had 2 visits). The MCP sign was observed in 33 (54.1%) premutation carries and Pallidal T2-abnormalities was detected in 2 controls (22%) and 11 premutation carriers (18%) of this cohort. We first tested the effect of pallidal T2-abnormalities on iron content including both controls and premutation carriers. Since T2-hyperintensities in the central region could indicate iron depletion due to gliosis and neurodegeneration, iron content in the globus pallidus was estimated using both average iron concentration (i.e., QSM mean) and iron variability (i.e., QSM CV). The pallidal T2-abnormalities demonstrated significant associations with accelerated decrease with age in QSM mean (age-by-pallidal T2-abnormalities: β = −0.97, SE = 0.37, *p* = 0.011), and accelerated increase with age in QSM CV (β = 0.024, SE = 0.006, *p* < 0.001) in the globus pallidus (Table 3; Figures 3A,B). We then examined the effect of pallidal and MCP T2-abnormalities on iron content in the globus pallidus in 61 premutation carriers. Neither sign individually showed a significant main effect on QSM mean or CV at FDR <0.05. However, the presence of both signs was associated with increased QSM CV in the globus pallidus (β = 0.26, SE = 0.08, *p* = 0.002) (Table 4; Figures 3C,D).

**Table 3:** The effect of pallidal abnormal T2-signals on iron content in the globus pallidus and dentate nucleus in controls and premutation carriers.

**Comparisons**	**β**	**SE**	***P***	**FDR**
**Globus pallidus QSM mean (ppm) (N** **=** **70)**				
Pallidus	4.31	2.49	0.09	0.11
Age-by-pallidus	−0.97	0.37	0.011^*^	0.018
**Globus pallidus QSM CV (N** **=** **70)**				
Pallidus	0.031	0.040	0.45	0.45
Age-by-pallidus	0.024	0.006	<0.001^*^	0.001
**Dentate nucleus QSM mean (ppm) (N** **=** **67)**				
PM vs. NC	8.19	2.96	0.007^*^	0.018

* *Significant at FDR 0.05*.

*SE, standard error; FDR, false discovery rate; QSM, quantitative susceptibility mapping; ppm, parts per million; CV, coefficient of variation; PM, premutation carriers; NC, normal controls*.

**Table 4:** The effect of T2-abnormalities in the MCP and globus pallidus on iron content in the globus pallidus in premutation carriers (*N* = 61).

**Comparisons**	**β**	**SE**	***P***	**FDR**
**Globus pallidus QSM mean (ppm)**				
Pallidus	7.56	3.67	0.045	0.09
MCP	2.73	1.92	0.16	0.19
Age-by-MCP-by-pallidus	−1.66	0.76	0.033	0.09
**Globus pallidus QSM CV**				
Pallidus	−0.044	0.065	0.51	0.51
MCP	−0.056	0.032	0.09	0.14
MCP-by-pallidus	0.26	0.08	0.002^*^	0.012

* *Significant at FDR 0.05*.

*SE, standard error; FDR, false discovery rate; QSM, quantitative susceptibility mapping; ppm, parts per million; CV, coefficient of variation; MCP, middle cerebellar peduncle*.

### Iron Accumulates in the Dentate Nucleus in the Premutation Group

Controlling for age, significant iron accumulation was observed in the dentate nucleus in the premutation group compared with controls (β = 8.19, SE = 2.96, *p* = 0.007) (Table 3). Three premutation carriers were excluded from the analyses because of incomplete coverage of the dentate nucleus in the QSM scans. Note that dentate nucleus volume, showing a strong positive correlation with mean QSM after age-adjustment (β = 12.7, SE = 2.48, *p* < 0.001), was not included as a covariate, as the potential effect of the same amount of iron deposited in a smaller structure due to atrophy was absent. Controlling for age, neither MCP nor pallidal T2-abnormalities showed a significant effect on measures of the dentate nucleus. Also controlling for age, among 58 premutation carriers, there were negative correlations of CGG repeat length with volume (β = −0.015, SE = 0.003 mm^3^, *p* < 0.001) and QSM mean (β = −0.16, SE = 0.08, *p* = 0.045) of the dentate nucleus (Figures 3E,F).

## Discussion

The results of this study demonstrate significant associations of the MCP sign with FXTAS motor and executive functioning and that hyperintense T2 signals in the globus pallidus, especially seen in combination with MCP changes, may contribute to accelerated deterioration of executive functioning in a subset of male *FMR1* premutation carriers. Iron content analysis revealed significant iron accumulation in the dentate nucleus in the premutation carriers and the association of pallidal T2-abnormalities with age-related, accelerated iron depletion and variability in the globus pallidus. We also observed significant associations of the MCP sign and measures of dentate nucleus with CGG repeat length, but not between pallidal measures and *FMR1* molecular markers.

As a key radiological biomarker of FXTAS, the MCP sign, demonstrated significant associations with intention tremor, cerebellar ataxia, and impaired executive functioning (Table 2), underscoring its central role in FXTAS pathology. In the current cohort of 374 males aged 8–85 years, the youngest man showing the MCP sign was a 50-year-old premutation carrier while no controls showed the MCP sign. The youngest premutation carrier showing pallidal T2-abnormalities was 47 years old, and the youngest control was 53 years old. Controlling for age, in 257 men aged ≥ 45 years, the prevalence of pallidal T2-abnormalities at their most recent visits was not significantly higher in premutation carriers (25.1%) than among controls (13.4%). In contrast, 52% premutation carriers but no controls showed the MCP sign. Other findings of interest were the appearance of the pallidal sign in 13.4% of controls, which may be a function of age. We know of no other data that report this sign in association with aging. The prevalence of the pallidal sign was approximately equivalent for carriers and controls, suggesting that for carriers, it is not a biomarker of a discrete neurodegenerative disorder.

The pattern of T2-signal changes in the globus pallidus observed in the current study resembles the eye-of-the-tiger sign, the neuroradiologic marker of pantothenate kinase-associated neurodegeneration (PKAN). PKAN is a rare inherited movement disorder (prevalence between 1:0.4 million and 1:1.5 million) (51) that primarily affects children and young adults (52). Eye-of-the-tiger sign, which is associated with iron depositions, is characterized by bilaterally hypointense T2-signals in the globus pallidus, surrounding a hyperintense central region marked by gliosis, neurodegeneration, and edema (53). In our participants with pallidal T2-abnormalities, most of them showed pallidal hyperintense signals totally surrounded by hypointense signals. However, in some participants with relatively large regions of pallidal T2-hyperintensities, the hyperintense regions may not be completely surrounded by hypointense regions. In addition, pallidal T2-abnormalities did not show a significant detrimental effect on motor, executive, or emotional functioning in older controls and premutation carriers. However, pallidal T2-abnormalities and the MCP sign, when seen in *combination*, revealed greater impairment of executive functioning (Table 2; Figure 2) in premutation carriers with both signs, compared to carriers with only one or neither of the signs, suggesting a synergistic interaction between the two regions.

These findings are consistent with the functions performed by the cerebellum and globus pallidus, which are part of the basal ganglia-cerebellar-cerebral cortical networks important for motor, cognitive, and emotional processing (21). However, although global psychological symptoms and depression were significantly increased in the premutation group compared with the control group after age-adjustment, significant associations were not observed with either pallidal T2-abnormalities or MCP sign. This could suggest low sensitivities of pallidal T2-abnormalities and MCP sign as indices of impaired emotional processing, or the critical involvement of other brain areas, such as the hippocampus and amygdala, that have been previously reported (54–56).

We further investigated the effect of MCP and pallidal T2-abnormalities on iron content in the dentate nucleus and globus pallidus. To the best of our knowledge, the associations of MRI or neuropathological changes in the MCP with brain iron depositions have not previously been reported in FXTAS. Quantified using QSM, pallidal T2-abnormalities were correlated with accelerated iron depletion and variability with age in the globus pallidus, but not the dentate nucleus, for both premutation carriers and controls (Figures 3A,B; Table 3). Iron accumulation in the globus pallidus did not differ among the groups, in contrast to iron content in the dentate nucleus, which was significantly higher among premutation carriers than controls. The association between pallidal T2-abnormalities and iron content was consistent with pathological findings of the eye-of-the-tiger sign in PKAN—a symmetrical focal region of gliosis, neurodegeneration, and edema with diamagnetic properties leading to *reduced* QSM values, surrounded by regions of paramagnetic iron depositions causing *increased* QSM values (53, 57). The potential presence of diamagnetic calcification could also reduce QSM values in that region, resulting in increased iron content variability and age-related reduction in the averaged QSM values in the globus pallidus for those with pallidal T2-abnormalities. In the current study, a pallidus-by-MCP interaction was also found in the globus pallidus, indicating increased iron variability among premutation carriers with both signs, when compared to the remaining premutation carriers (Figure 3D; Table 4). In contrast, no significant effect of pallidal or MCP T2-abnormalities on iron content of the dentate nucleus was found.

Distinct molecular mechanisms may underlie structural changes in the cerebellum and globus pallidus, supported by the selective associations of CGG repeat length with cerebellar measures, namely the MCP sign (positive, *p* < 0.001), and anatomic volume and iron accumulation in the dentate nucleus (negative, *p* < 0.001 and 0.045, respectively), but not with measures of the globus pallidus (i.e., T2-abnormalities and iron content). Interestingly, the MCP sign was observed in 65% of premutation carriers with CGG repeat length >75 at their last visits, but not among controls or premutation carriers with CGG repeat length ≤ 75 at any visits. This suggests a high vulnerability of the cerebellum among individuals with CGG repeat expansions in the medium to high premutation range.

Specific mechanisms of how CGG repeat expansions in the premutation range (55–200) are associated with neuropathology are unknown. Mitochondrial dysfunction mediated by dysregulated iron metabolism has been observed during aging (58), as well as in fibroblasts from premutation carriers with and without FXTAS (59). Postmortem examinations provided supporting evidence of dysregulated iron transport in the putamen and choroid plexus in brains of individuals with FXTAS (17, 18). Alternatively, microangiopathy, recently proposed as a feature of FXTAS (60), might lead to iron accumulation and neurodegeneration in the subcortical nuclei.

We did not find an association of pallidal T2-abnormalities with major motor or psychiatric impairment in older controls or premutation carriers with the sign (age ≥ 45). Rather, iron accumulation and neurodegeneration underlying pallidal T2-abnormalities might interact with pathology associated with the MCP sign, accelerating cognitive impairment. Iron plays a critical role in developing brains, where it acts as a vital metabolic cofactor for core processes such as myelination, dendritogenesis, and neurotransmitter synthesis. The globus pallidus, maintaining a particularly active metabolism during childhood, is vulnerable to iron dysregulation (61). During aging, however, the brain may retain sufficient capacity to compensate for pallidal neurodegeneration. Although speculative, our findings suggest that neuropathology in the globus pallidus could be compensated for by the cerebellum. However, structural damage in both globus pallidus and cerebellum, two critical nodes in the basal ganglia-cerebellar-cerebral cortical networks, are particularly detrimental to cognitive functioning, as evidenced by the significant interaction between pallidus and MCP abnormalities on executive functioning. Our findings further suggest that the interaction between pallidal and MCP abnormalities may contribute to heterogeneous clinical presentations of FXTAS. The pallidal sign may be relevant for identifying patients with FXTAS who have the risk of experiencing accelerated cognitive decline as well as for stratifying vulnerable patients for potential targeted treatments such as neurosteroids that promote regeneration and repair (62–64). Further studies are needed to establish the links among *FMR1* premutation, iron dysregulation, and the occurrence of pallidal T2-abnormalities.

The high rates of intention tremor (21.4%) and cerebellar ataxia (10.0%) among the controls, who were recruited from pedigrees or local community, were unexpected. For all participants including the controls, we conducted detailed, often blinded and video-taped neurological examinations to detect tremor and ataxia. The severity of tremor or ataxia was mild for the 18 controls who showed either cerebellar ataxia and/or intention tremor, suggesting that these may be associated with aging rather than any type of disease process.

The prevalence of intention tremor in the general population is unclear but may be estimated from that of essential tremor, which may lie on the same continuum of severity as intention tremor (65). Epidemiological studies have found that essential tremor is among the most common movement disorders in older adults (aged ≥65), with a prevalence in the general population similar to that for Alzheimer's disease (median 4.8%) (66). However, mild postural and kinetic tremor associated with aging has been observed in almost all (96%) normal controls (defined as not fulfilling criteria for essential tremor, aged 19–93), indicating the challenges in distinguishing normal and pathological tremor (67), and the potential underestimate of tremor prevalence. The population-based prevalence for late-onset cerebellar ataxia was estimated to be 10.2/100,000 including both the idiopathic and inherited forms in southeast Wales (68), a value that is clearly much lower than we observed in our controls. It is possible that individuals with mild tremor or ataxia are more likely to participate in clinical research, and this may have provided us an opportunity to examine the effect of pallidal T2-abnormalities on tremor and ataxia. We are currently using more objective measures of tremor and ataxia to improve assessment precision. The significantly lower depression scores in controls with the pallidal sign compared with controls without the sign (Supplementary Table 1) is intriguing. However, only 8/11 controls with the pallidal sign had available depression scores, so the sample was too small to draw a firm conclusion about this. Further investigations in larger samples are needed to confirm the finding, as well as the clinical significance of the pallidal T2-abnormalities in older controls.

A strength of this study was the large number of MRI scans; however, only 48.2% of participants aged ≥45 had more than one clinical visit, and hence the results were based on a combination of cross-sectional and longitudinal data. QSM data were available for only 9 controls, which most likely diminished the statistical power to detect a difference in pallidal iron concentration between control and premutation groups. Given that the present sample in a study of an X-linked condition consisted entirely of males, it is important that future research repeat these analyses with females.

## Conclusions

We report the observation of hyperintense T2-signals in the MCP and globus pallidus among older (age ≥45) premutation carriers (MCP 53%, pallidus 25.1%, sample prevalence) and healthy controls (MCP 0%, pallidal 13.4%, sample prevalence). The MCP sign demonstrated a significant negative effect on executive functioning, as well as on kinetic tremor and cerebellar ataxia and the occurrence of combined MCP and pallidal T2-abnormalities was associated with significantly worse executive function in premutation carriers. In contrast, pallidal abnormalities alone, were not associated with core FXTAS motor symptoms such as tremor and ataxia in either controls or premutation carriers. Magnetic susceptibility analysis using QSM revealed significant iron accumulation in the dentate nucleus in premutation carriers whereas pallidal T2-abnormalities were associated with age-related, accelerated iron depletion and variability in the globus pallidus and having T2-abnormalities in both globus pallidus and MCP further increased iron variability. Distinct molecular mechanisms may underlie structural changes in the cerebellum and globus pallidus in FXTAS, evident in significant correlations between CGG repeat length and cerebellar abnormalities, but not between *FMR1* molecular measures and the globus pallidus. Underlying neural structural pathology reflected in the observed interaction between MCP and pallidal T2-signal changes may contribute to heterogeneous clinical manifestations of FXTAS.

## Data Availability Statement

The raw data supporting the conclusions of this article will be made available by the authors, without undue reservation.

## Ethics Statement

The studies involving human participants were reviewed and approved by University of California Davis Institutional Review Board. Written informed consent to participate in this study was provided by the participants and/or the participants' legal guardian/next of kin.

## Author Contributions

DH, SR, and RH acquired funding for the research project and supervised the recruitment of research participants and data acquisition. JW conceived the study and executed statistical analysis. JW, JG, DH, SR, and RH designed the study. JW, DP, and HW performed MRI image processing and data generation. FT generated *FMR1* molecular data. JW and KK designed statistical analysis and performed data interpretation. JW and JG wrote the first draft of the manuscript. All authors have reviewed and criticized the manuscript. All authors contributed to the article and approved the submitted version.

## Funding

This project was supported by NIH grants NS110100 (to SR and DH), MH078041 (to DH and SR), HD036071 (to RH), private donors, and the MIND Institute Intellectual and Developmental Disabilities Research Center P50 HD103526.

## Conflict of Interest

Author RH had received funding from Zynerba, Ovid, and the Azrieli Foundation to carry out treatment studies in fragile X syndrome, and had also consulted with Zynerba and Fulcrum regarding treatment studies in fragile X syndrome. Author DH had received funding from Novartis, Roche, Zynerba, and Seaside Therapeutics for consultation regarding treatment studies in fragile X syndrome. Author FT had received funding from Zynerba and the Azrieli Foundation to carry out molecular studies in fragile X syndrome. The remaining authors declare that the research was conducted in the absence of any commercial or financial relationships that could be construed as a potential conflict of interest.

## Publisher's Note

All claims expressed in this article are solely those of the authors and do not necessarily represent those of their affiliated organizations, or those of the publisher, the editors and the reviewers. Any product that may be evaluated in this article, or claim that may be made by its manufacturer, is not guaranteed or endorsed by the publisher.
