# Supplementary material for: Clinical and Molecular Correlates of Abnormal Changes in the Cerebellum and Globus Pallidus in Fragile X Premutation
Source: Front Neurol. 2022 Feb 8;13:797649. doi: 10.3389/fneur.2022.797649 (PMC8863211; doi:10.3389/fneur.2022.797649)
Supplement: Supplementary file 1 [file Table_1.pdf]

Supplementary Materials for Clinical and Molecular Correlates of Abnormal Changes in the Cerebellum and Globus Pallidus in Fragile X Premutation

Supplementary Table 1 Comparisons of controls with and without pallidal T2-abnormalities at last visits.

Measurements	No Pallidal Sign	With Pallidal Sign	Comparisons	95% CI	*P* Values	FDR
Controls						
Age: mean (SD) [N]	62.9 (8.2) [71]	68.5 (8.0) [11]	t = 2.18	0.08, 11.22	0.047	0.19
Intention tremor: % [N]	21.3 [61]	22.2 [9]	OR = 0.82	0.15, 4.66	0.83	0.95
Cerebellar ataxia: % [N]	9.8 [61]	11.1 [9]	OR = 0.97	0.10, 9.38	0.98	0.98
BDS: mean (SD) [N]	22.0 (3.1) [60]	22.88 (2.9) [8]	β = 1.24	-1.01, 3.49	0.28	0.56
Psych. symptoms: mean (SD) [N]	53.3 (11.4) [53]	46.1 (7.5) [8]	β = -5.91	-13.7, 1.88	0.14	0.37
Depression: mean (SD) [N]	55.2 (10.8) [53]	43.3 (6.0) [8]	β = -10.9	3.46, 18.3	0.006	0.048
CGG repeat: mean (SD) [N]	29.0 (4.3) [69]	28.3 (6.5) [11]	t = -0.34	-5.14, 3.74	0.74	0.95
FMR1 mRNA: mean (SD) [N]	1.35 (0.37) [68]	1.25 (0.47) [11]	t = -0.66	-0.42, 0.22	0.52	0.83

CI, confidence interval; FDR, false discovery rate; SD, standard deviation; OR, odds ratio; BDS, Behavioral Dyscontrol Scale; *FMR1*, fragile X mental retardation 1
